# Transcriptome sequencing and screening of genes related to glucose availability in *Schizosaccharomyces pombe* by RNA-seq analysis

**DOI:** 10.1590/1678-4685-GMB-2020-0245

**Published:** 2021-08-27

**Authors:** Çağatay Tarhan, Özgür Çakır

**Affiliations:** 1 Istanbul University, Faculty of Science, Department of Molecular Biology and Genetics, Istanbul, Turkey.

**Keywords:** Glucose availability, Schizosaccharomyces pombe, aging, gene regulation, RNA-Seq

## Abstract

While calorie restriction is the most used experimental intervention to increase lifespan in numerous model organisms, increasing evidence suggests that excess glucose leads to decreased lifespan in various organisms. To fully understand the molecular basis of the pro-aging effect of glucose, it is still important to discover genetic interactions, gene expression patterns, and molecular responses depending on glucose availability. Here, we compared the gene expression profiles in *Schizosaccharomyces pombe* mid-log-phase cells grown in three different Synthetic Dextrose media with 3%, 5%, and 8% glucose, using the RNA sequencing method. Expression patterns of genes that function in carbohydrate metabolism were downregulated as expected, and these genes were downregulated in line with the increase in glucose content. Significant and consistent changes in the expression were observed such as genes that encoding retrotransposable elements, heat shock proteins, glutathione S-transferase, cell agglutination protein, and conserved fungal proteins. We group some genes that function together in the transcription process and mitotic regulation, which have recently been associated with glucose availability. Our results shed light on the relationship between excess glucose, diverse cellular processes, and aging.

## Introduction

The nutritional condition is one of the most important determinants of cellular and organismal life-span. It has been shown in a wide range of species that, while overnutrition accelerates aging, nutritional restriction, which is also called calorie restriction, delays it (Reverter-Branchat et al., 2004). Once it was realized that limited nutrition not only increases longevity but prevents or retards some age-related diseases (Anderson and Weindruch, 2012), the focus of aging studies has shifted to uncover the underlying genetic and molecular mechanisms of calorie restriction and aging. Mainly two approaches are used to understand these mechanisms. One of them, single-gene mutations or overexpression, has been used to identify the genes whose overexpression or deletion mimic the effect of calorie restriction, or that have a considerable effect on longevity by some other mechanisms. In this regard, although they mostly consider the protein coding regions and exclude the role of noncoding and regulatory regions, many attempts have been made in a variety of organisms such as *Escherichia coli*, *Saccharomyces cerevisiae*, *Drosophila melanogaster*, *Caenorhabditis elegans* and mice (Fontana et al., 2010, Rallis et al., 2013, Dancy et al., 2014, Sideri et al., 2014, Boehm et al., 2016). 

Another approach is to perform whole-genome expression profiling when cells or organisms are grown in poor or rich nutritional conditions, or when they are in the natural aging process (Rallis et al., 2014, Baumgart et al., 2016, Hall et al., 2017). This method is quite fruitful because it promises to gain more integrative insights and to find new genes, interactions or regulatory mechanisms that contribute to the process of natural aging or adaptation to an experimental condition. 

Both approaches have revealed important insights into the relationship between nutrition and some well-known fundamental cellular pathways, such as insulin/insulin-like growth factor 1 (IGF-1) signaling (IIS), the Target of Rapamycin (TOR) and the energy-sensing pathway AMP-activated protein kinase (AMPK). Their downstream elements and interactions with other cellular processes have also been identified. For example, it is well established in various organisms that the IIS pathway is activated in response to nutrients and acts in part through mTOR to impact lifespan (Papadopoli et al., 2019). On the other hand, amino acid metabolism, oxygen, various stressors or energy input affect mTOR activity and thereby regulates other metabolic pathways that control cell growth. Activation of the mTOR pathway leads to the inhibition of autophagy, which is an important cellular self-degradative process in the determination of cell fate, aging, or disease development. AMPK is activated by changes in the cellular AMP/ATP ratio and the increasing activity has been shown to extend lifespan in various organisms (Mair et al., 2011, Wierman et al., 2017). 

Since glucose is the common source of energy in most prokaryotes and eukaryotes, its availability directly affects these and other pathways thereby organismal aging. Manipulating the glucose concentration in food or media, many researchers have shown alteration and/or restructuring in the activity of these pathways and the expression levels of their members. For example, elevated glucose in diet shortened lifespan in *C. elegans* by downregulating proteins such as AMPK, FOXO, and glyoxalase (Lee et al., 2009) In *S. cerevisiae*, glucose limiting leads to 35% extension in lifespan (Lin *et al*., 2000), and lowering the glucose concentration from 2% to 0.1% prolonged yeast lifespan up to 50% (Jiang et al., 2000). On the other hand, increasing glucose concentration in the medium from 2% to 10% induced superoxide ion production, which causes the inhibition of growth arrest and led to DNA replication stress in *S. cerevisiae* (Weinberger et al., 2010). After determining that glucose shortened lifespan in a dose-dependent manner in *S. pombe*, Roux et al. (2009) showed that not only the glucose metabolism but also the extracellular glucose signaling might be responsible for decreasing chronological lifespan. They found that the deletion of *git3* ^*+*^ , which encodes a G protein-coupled receptor protein that senses environmental glucose, causes an effect that mimics calorie restriction (Roux *et al*., 2009). Chen and Runge (2009) suggested that glucose shortens the lifespan of *S. pombe* cells partly because remaining glucose signals cells to grow and thereby prevents them to be arrested in a quiescent state. Besides, they showed that the deletion of serine/threonine-specific protein kinase (AKT) orthologs *sck1* ^*+*^ and *sck2* ^*+*^ , caused a significant lifespan extension especially in the presence of excess glucose. This finding suggests that these genes efficiently function in the control of longevity (Chen and Runge, 2009). Although it is not well established that glucose accelerates aging in humans and other mammals, some studies have made significant contributions to answering this question. For example, excess glucose consumption increased the percentage of polyploid β-cells, and this increase may reflect premature aging of these β-cells in mice (White et al., 1985). More recently, Mortuza et al. (2013) reported that excess glucose accelerates aging in different types of human endothelial cells by decreased activity of sirtuins which are mediated through FOXO1. In another study, Zhang et al. (2017) showed that mesenchymal stem cells aging-induced when the cells treated with 11 or 22 mM glucose for 14 days, and they suggested that the Akt/mTOR signaling pathway acts as the primary mediator in the aging process induced by excess glucose (Zhang *et al*., 2017). These and other attempts have yielded important insights into the role of glucose and energy metabolism in the process of aging. Yet there is still more to discover by testing different conditions and media, investigating different time intervals, using active substances, mutant strains or experimental combinations in the manipulation of the aging process of an organism.

In this study, to evaluate the transcriptome-wide changes in the presence of excess glucose, we performed a genome-wide mRNA expression profiling (RNA-seq) analysis in wild-type *Schizosaccharomyces pombe* which has become a popular model organism in aging studies in recent years. We used three Synthetic Dextrose (SD) media which contain 3%, 5%, and 8% glucose, represented by T1, T2, and T3 respectively. As glucose concentration increased, the expression of genes associated with carbohydrate metabolism was downregulated as expected. We observed a marked and consistent change in the expression of the genes encoding retrotransposable elements, heat shock proteins, glutathione S-transferase, cell agglutination protein, as well as genes encoding conserved fungal proteins. Besides, we evaluated the changes in the expression of a group of genes (e.g. transcription coactivators or mitotic progression regulators) that have recently been associated with glucose availability and metabolism. Our results contribute to correlate diverse cellular processes with excess glucose availability and its pro-aging effects. 

## Material and Methods

### Yeast strain, media, and growth conditions

The wild type strain of *Schizosaccharomyces pombe* (972 h^-^) was used in all experiments. Because it was reported that the Synthetic Dextrose (SD) media were an appropriate condition for lifespan experiments and cells grown in SD with excess glucose showed the evolutionarily conserved response to lifespan (Chen and Runge, 2009), we used this medium. Cells from a single colony were inoculated in three different SD media with 3%, 5%, and 8% glucose, which are represented by T1, T2, and T3 respectively. Initial cell density was 5 × 10^4^ cells/ml and cells were grown up to mid-log phase in 25 ml of medium in a 100 ml flask at 30°C with orbital shaking at 180 rpm. 

### Total RNA isolation

Total RNAs were isolated from mid-log cells using GeneJET RNA Purification Kit (Thermo Fisher Scientific) following the manufacturer’s instructions for each sample. The purity and quantity of RNAs were measured using a Nanodrop 2000 spectrophotometer (Nanodrop Technologies, USA). Total RNAs were then used in further analysis. Three replicates were used for each sample and replicates were then pooled for sequencing according to their concentrations.

### Library preparation and sequencing

Fragmentation, adapter binding, cDNA library preparation, and RNA sequencing were performed by Beijing Genome Institute (Shenzhen, China). Briefly, the RNAs were fragmented in fragmentation buffer. After this treatment, cDNA synthesis was done by using the fragments as templates with random hexamer primers. Adapters were ligated to short fragments, then, PCR was performed. Lastly, Illumina HiSeq 2000 was used for paired end sequencing.

### Bioinformatic analysis of sequence data

Firstly, the raw reads of low quality were eliminated before any advanced analysis. Filtering was done to eliminate adapter sequences (SOAPnuke software, ver. 1.5.6, http://soap.genomics.org.cn/), sequences with a high content of unknown bases more than 10% and sequences with low-quality scores (less than 10). Residual reads were accepted as clean reads. Clean reads were then aligned to the *Schizosaccharomyces pombe* genome and genes using BWA (Burrows-Wheeler Aligner, ver 0.7.12, (Li and Durbin, 2010)) and BOWTIE (ver.1.1.2) (Langmead et al., 2009) software, respectively. The expression of aligned genes and isoforms was then measured and quantified by RSEM software package (ver. 1.2.25) (Li and Dewey, 2011). RSEM takes into account the maximum likelihood abundance estimates by the Expectation-Maximization (EM) algorithm as its statistical model, including the modeling of paired-end (PE) and variable-length reads, fragment length distributions, and quality scores, to specify the transcripts that are isoforms of the same gene. The expression levels were calculated by using the FPKM (fragments per kilobase per million reads) and Tophat method was used to determine the alternative splicing event detection (2.1.0) (Trapnell et al., 2009). We did not want to determine a random FPKM value to determine whether a transcript was expressed. However, analysis of all transcripts with expression levels greater than zero must include FPKM values very close to zero. Therefore, we chose 0.005 FPKM as the lower limit for the subsequent analysis. Differentially expressed genes were screened according to the Poisson distribution method and NOIseq package (FDR<0.001 and log2 [sample1/sample2] >1) (ver. 2.14.1) (Tarazona et al., 2011). For gene ontology analysis, differentially expressed genes first mapped to GO terms in gene ontology database (www.geneontology.org). The gene numbers for each GO term were calculated (Corrected p value ≤ 0.05 as threshold). The mapped genes were classified according to three different categories, molecular function, cellular component, and biological process. The figures are drawn with respect to significantly enriched GO terms. The functional classification was done by using WEGO software (Ye et al., 2006). Lastly, KEGG was used to analyze significantly enriched pathways between samples (Qvalue≤0.05) (Kanehisa et al., 2008). KEGG enrichment analysis was done by the algoritm developed by BGI. Data were submitted to https://www.ncbi.nlm.nih.gov/geo/ (Accession number GSE159723).

## Results

**Figure 1 -:**
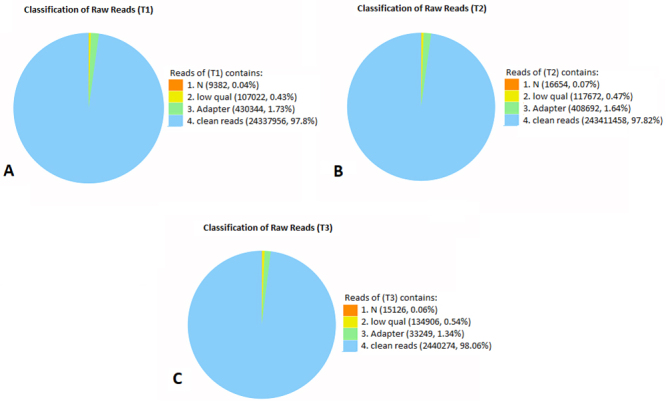
Classification of raw reads in samples A) T1, B) T2, C) T3.

The RNAs from the cells grown in SD medium with 3%, 5%, and 8% glucose were sequenced on Illumina Hiseq 2000 platform. A total of 74,654,458 raw reads were obtained from the sequencing of three different libraries. Quality control was done by filtering the raw data (Figure 1). The adapter sequences and the sequences which contain more than 10% unknown bases and low-quality score sequences were eliminated. The number for clean reads was 73,082,162. While genome mapping rates were 94.83, 94.85 and 94.51, gene mapping rates were 90.28, 90.92 and 89.72 % for T1, T2 and T3 samples, respectively (Table 1). Slightly over 5000 genes were found to be expressed in each sample. For T1, 78.50% of the reads were perfectly matched with the reference genome while 76.20% of the reads were matched perfectly to reference genes. The perfect matches on a reference genome for T2 and T3 were 79.10 and 79.24 respectively. Also, perfect matches on reference genes for T2 and T3 were 77.13 and 76.47 respectively. The statistics of sequencing were shown in Table 1.

**Table 1 -:** Statistics of raw data belonging to different samples.

	Map to Genome	Reads Number	Percent	Map to Gene	Reads Number	Percent
T1	Total Reads	24,337,956	100.00%	Total Reads	24,337,956	100.00%
	Total BasePairs	2,433,795,600	100.00%	Total BasePairs	2,433,795,600	100.00%
	Total Mapped Reads	23,080,889	94.83%	Total Mapped Reads	21,971,788	90.28%
	Perfect Match	19,104,654	78.50%	Perfect Match	18,544,717	76.20%
	Mismatch	3,976,235	16.34%	Mismatch	3,427,071	14.08%
	Unique Match	22,343,264	91.80%	Unique Match	18,848,762	77.45%
	Multi-position Match	737,625	3.03%	Multi-position Match	3,123,026	12.83%
	Total Unmapped Reads	1,257,067	5.17%	Total Unmapped Reads	2,366,166	9.72%
T2	Total Reads	24,341,458	100.00%	Total Reads	24,341,458	100.00%
	Total BasePairs	2,434,145,800	100.00%	Total BasePairs	2,434,145,800	100.00%
	Total Mapped Reads	23,086,706	94.85%	Total Mapped Reads	22,130,242	90.92%
	Perfect Match	19,233,184	79.01%	Perfect Match	18,775,050	77.13%
	Mismatch	3,853,522	15.83%	Mismatch	3,355,187	13.78%
	Unique Match	22,362,920	91.87%	Unique Match	19,150,654	78.68%
	Multi-position Match	723,786	2.97%	Multi-position Match	2,979,588	12.24%
	Total Unmapped Reads	1,254,752	5.15%	Total Unmapped Reads	2,211,214	9.08%
T3	Total Reads	24,402,748	100.00%	Total Reads	24,402,748	100.00%
	Total BasePairs	2,440,274,800	100.00%	Total BasePairs	2,440,274,800	100.00%
	Total Mapped Reads	23,062,621	94.51%	Total Mapped Reads	21,895,314	89.72%
	Perfect Match	19,336,002	79.24%	Perfect Match	18,660,950	76.47%
	Mismatch	3,726,619	15.27%	Mismatch	3,234,364	13.25%
	Unique Match	22,080,264	90.48%	Unique Match	18,925,418	77.55%
	Multi-position Match	982,357	4.03%	Multi-position Match	2,969,896	12.17%
	Total Unmapped Reads	1,340,127	5.49%	Total Unmapped Reads	2,507,432	10.28%

**Figure 2 -:**
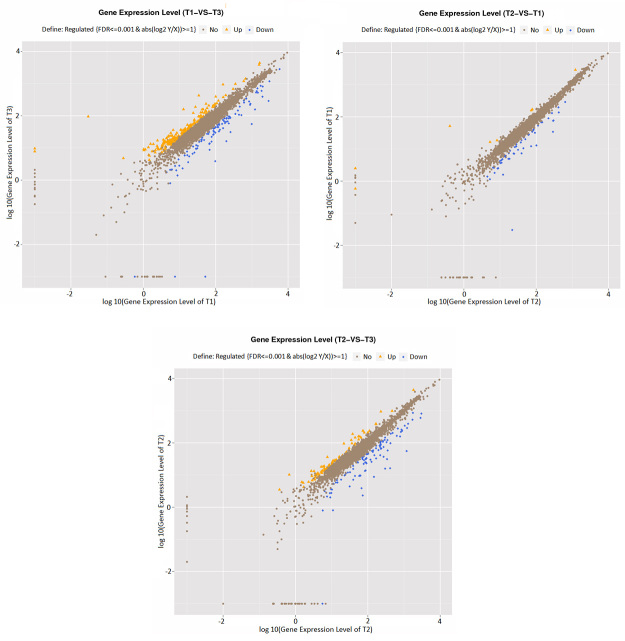
Differential expression levels of T1, T2, and T3 samples. Yellow and blue color represent differentially expressed genes, Brown color represents the genes whose expression does not differ in samples.

**Figure 3 -:**
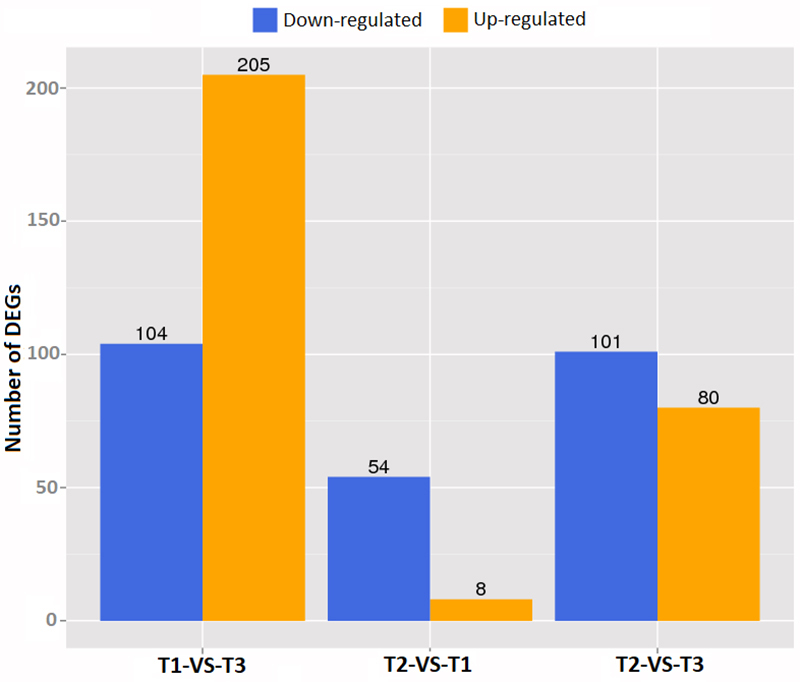
The gene numbers of differentially expressed genes. Blue color represents downregulation and yellow color represents upregulation.

To investigate the effect of different glucose concentrations on gene expression profile, we analyzed the T1, T2, and T3 samples with respect to differentially expressed genes (Figure 2). The number of commonly expressed genes and only the number of genes whose expression significantly differentiated were considered and given for each condition. Accordingly, 5061 genes were expressed commonly in both T1 and T2 samples, while only 8 genes were expressed in T1 and 21 genes in T2. When we compared the T1 and T3, 5045 were in common, 24 were expressed in T1 and 13 were expressed in T3. Lastly, 5049 were expressed in both T2 and T3 while 33 were expressed only in T2 and 9 in T3. Glucose concentration significantly affected gene expression between samples. When we compared T1 and T3, 104 genes were downregulated (FDR≤0.001, log2 ratio≤1) and 205 genes were found to be upregulated (FDR≤0.001, log2 ratio≥1) (Figure 3). For T2 and T1 comparison, there were 54 genes downregulated (FDR≤0.001, log2 ratio≤1) and 8 genes upregulated (FDR≤0.001, log2 ratio≥1). The upregulated genes were 80 (FDR≤0.001, log2 ratio≥1) and downregulated ones were 101 (FDR≤0.001, log2 ratio≤1) when T2 and T3 were compared.

After gene ontology annotations to reveal the distribution of gene functions, functional classification was performed for differentially expressed genes. The gene ontology analysis showed that the metabolic process (GO ID: 0008152, number of genes: 38) and cellular process (GO ID: 0009987, number of genes: 31) were the categories with the highest gene numbers in biological process in the comparison between T1 and T3. In the cellular component class in the same comparison, cell (GO ID: 0005623, number of genes: 21) and cell part (GO ID: 0044464, number of genes: 21) categories had the highest gene numbers. Lastly, in molecular function, binding (GO ID: 0005488, number of genes: 39) and catalytic activity (GO ID: 0003824, number of genes: 48) were prominent. 

For the comparison between T2 and T1, localization (GO ID: 0051179, number of genes: 5), metabolic process (GO ID: 0008152, number of genes: 7) for biological process class, cell (GO ID: 0005623, number of genes: 2), cell part (GO ID: 0044464, number of genes: 2) and membrane (GO ID: 0016020, number of genes: 3) for cellular component class, binding (GO ID: 0005488, number of genes: 6) and catalytic activity (GO ID: 0003824, number of genes: 10) for molecular function class had the highest gene numbers. 

**Figure 4 -:**
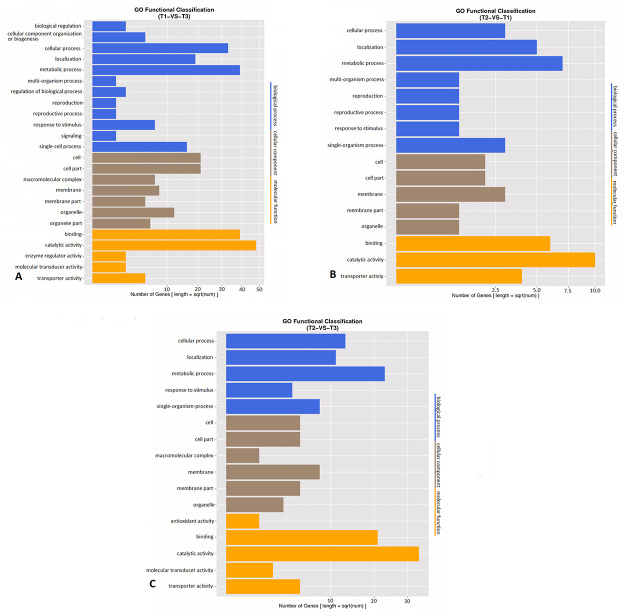
GO classification of differentially expressed genes between groups, A) T1 vs T3, B) T2 vs T1, C) T2 vs T3.

**Figure 5 -:**
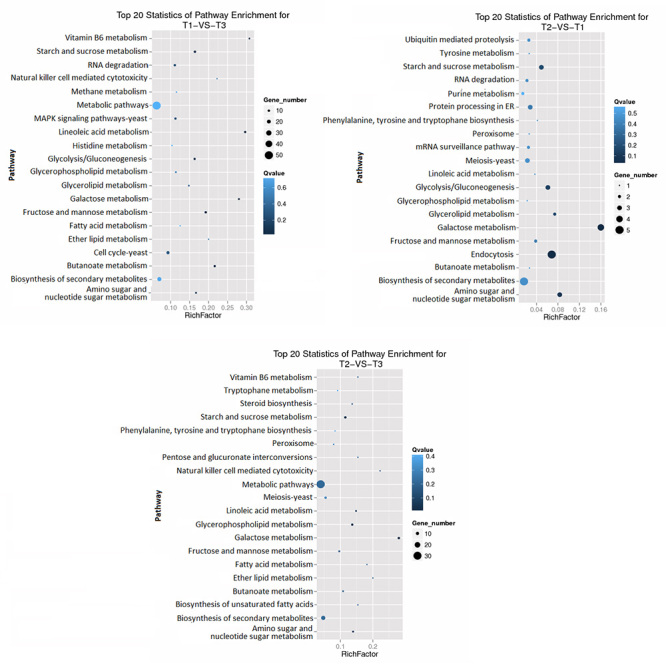
Chart of top 20 pathways between different treatment groups.

For the comparison between T2 and T3, cellular process (GO ID: 0009987, number of genes: 13), metabolic process (GO ID: 0008152, number of genes: 23) for biological process class, cell (GO ID: 0005623, number of genes: 5), cell part (GO ID: 0044464, number of genes: 5), membrane part (GO ID: 0044425, number of genes: 5) and membrane (GO ID: 0016020, numbero f genes: 8) for cellular component class, binding (GO ID: 0005488, number of genes: 21) and catalytic activity (GO ID: 0003824, number of genes: 34) for molecular function class had the highest gene numbers (Figure 4). Also, KEGG pathway enrichment analysis was done for differentially expressed genes between samples and the top 20 pathways are shown in Figure 5.

The quantitative change in the expression of individual genes depending on the glucose concentration in the medium is given in Table S1.

## Discussion

### Gene functions in carbohydrate metabolism

*gal1*^*+*^ , *gal7* ^*+*^ , and *gal10* ^*+*^ genes function in galactose utilization in *S. pombe*. Although the growth of *S. pombe* does not require galactose indispensably, galactosylation is crucial for some cellular processes including the maintenance of normal cell shape, drug tolerance, and nonsexual flocculation. (Takegawa and Matsuzawa, 2014 **)** To do this, at first galactose is transported into the cytosol and then the two enzymes, Gal1 and Gal7, involves in the conversion of galactose into UDP-galactose. Under glucose-limited conditions, Gal10 functions especially in galactosylation of cell-surface proteins (Matsuzawa *et al*. , 2011)**.** In our study, the expressions of *gal1* ^*+*^ , *gal7* ^*+*^ gal10^*+*^ are downregulated at elevated glucose levels. These results were consistent with the other studies. For example, it is reported that while the expression of *gal10* ^*+*^ is strongly upregulated by glucose repression, *gal10* ^*+*^ is repressed in glucose-rich medium in *S. pombe* (Suzuki et al., 2010)**.** In *S. cerevisiae*, glucose repression of GAL genes occurs through Mig1p global repressor protein (Nehlin et al., 1991). Besides**,** the transcription level of SPBPB2B2.11 which is predicted to encode a nucleotide-sugar 4,6-dehydratase enzyme was downregulated in the presence of 8% glucose when compare to SD with 5% glucose. This gene is located very close to the GAL genes and the expression pattern is similar to those of these genes regarding galactose presence in the culture medium (Matsuzawa *et al*., 2011).

The transcription level of invertase encoding *inv1* ^*+*^ and mitochondrial glycerol dehydrogenase encoding *gld1* ^*+*^ are also decreased significantly under glucose-rich conditions. It has been reported that the expression of these genes is downregulated when a high level of glucose is present in the culture medium. The fission yeast transcriptional regulator Scr1 mediates the repression of *inv1* ^*+*^ and *gld1* ^*+*^ (Tanaka et al., 1998, Matsuzawa et al., 2010). Similarly, the expression level of *gut*2^+^ which encodes the glycerol-3-phosphate dehydrogenase decreased consistently when glucose concentration was high. Although a direct link has not been shown between glucose availability and the *gut2* ^*+*^ expression in *S. pombe*, *Saccharomyces cerevisiae* ortholog of *gut2* ^*+*^ was shown to be subjected to glucose repression (Grauslund and Ronnow, 2000).

*S. pombe* has eight genes that encode the corresponding eight hexose transporters (*ght1* ^*+*^ to *ght8* ^*+*^ ) (Heiland et al., 2000). According to our results, two of them, *ght1* ^*+*^ and *ght5* ^*+*^ are subjected to glucose repression in both conditions. Kim et al., (2010) showed that all eight transporter genes are nonessential when the glucose concentration was 3% in culture medium (Kim *et al*., 2010)**.** It is also reported that the transcript level of the major hexose transporter *ght5* ^*+*^ and *ght1* ^*+*^ , which has a moderate affinity to glucose, was increased in the low-glucose medium. In the same study, it was also demonstrated that when the glucose concentration high, the *ght5* ^*+*^ expression was repressed by Scr1 which also represses *inv1* ^*+*^ transcription (Saitoh et al., 2015)**.** **S. pombe** genome encodes another Scr1-like protein, called Rsv1. This protein was shown to act as a transcriptional repressor and the deletion of *rsv1* ^*+*^ induced the genes that are involved in carbohydrate metabolism including *ght1* ^*+*^ (Mata et al., 2007)*.* In our study, when the glucose concentration in the medium was 8%, the expression level of *rsv1* ^*+*^ was 2,82 fold lower than that observed in SD with 5%. Taken together, our results showed that the expression levels of genes that function in carbohydrate metabolism consistently changed and regulated according to glucose content in the medium. When Vassiliadis and others performed a transcriptome analysis of preferential glucose utilization in *S. pombe*, they revealed that carbon metabolism pathways significantly upregulated in the glucose-deficient condition and they clarified that Scr1p regulates certain genes (e.g. *inv1+*, *ght5+*, and *gld1+*) involved in carbon metabolism, hexose uptake, gluconeogenesis, TCA cycle. However, in that study, Yeast Extract Supplemented (YES) medium was used (Vassiliadis *et al.*, 2019). Another genome-wide study, which also used YES as the medium, found that carbohydrate metabolism and meiotic differentiation were repressed in glucose rich medium (Malecki et al., 2016). Considering the similar results we obtained, our study also contributed to prove that SD medium could be used in longevity and energy metalobism related issues.

*gid2*^*+*^ is predicted to be the subunit of glucose-induced degradation deficient (GID) complex ubiquitin-protein ligase E3. In gluconeogenesis, fructose-1,6-bisphosphatase is expressed when there is no fermentable carbon source in medium, but in the presence of glucose, the enzyme undergoes a rapid degradation (Holzer, 1989). In *S. cerevisiae*, Gid2/Rmd5 is the *S. pombe gid2* ^*+*^ ortholog and responsible for the proteasomal degradation of the enzyme to block gluconeogenesis (Santt et al., 2008). Thus, the increase in *gid2* ^*+*^ expression in the T3 condition may indicate a similar regulation in *S. pombe*.

*agl1*+ functions in maltose utilization when especially carbon source in the medium is switched from glucose to maltose or glucose is completely depleted (Kato et al., 2013). In this study, its expression was downregulated in all tested conditions, implying that cells in that phase might still be using glucose and there was no need to induce *agl1*+ expression.

### Genes other than those that function in carbohydrate metabolism

While the increase in the expression of one of the two Tf2-type retrotransposable elements, SPAPB15E9.03c, in T2/T1 was 9,71 fold, the expression of SPAC26A3.13c upregulated 2,24 fold in T3 condition when compared to T2 condition. Although *Tf2* mRNA levels are not necessarily accompanied by a proportional change in mobilization (Murton et al., 2016), this expression pattern suggests an association between glucose availability and *Tf2* expression levels. On the other hand, Mallet et al. (2017) showed that antisense *Tf2* transcripts were maximally induced during meiotic division cycle, especially when sexual differentiation program was initiated. According to our results, the expression of *mat1-Mc* gene, which encodes a mating-type m specific polypeptide and controls the mating-type (Kelly et al., 1988) was markedly lower (-8.72 fold) in T2 condition when compared to T1. This change in the expression might have indicated that the sexual differentiation and some other dependent process halted in the presence of excess carbon source. It has been reported that the expression of this gene activated by Ste11p (Sugimoto et al., 1991) and it was shown in another study that starvation induces the *ste11* ^*+*^ transcript through decreased Protein Kinase A (PKA) activity (Valbuena and Moreno, 2010). On the other hand, it was shown that aforementioned Scr1p is required to repress sexual differentiation under glucose-sufficient conditions (Vassiliadis et al., 2019) Thus, it seems that, in the presence of excess glucose, activated PKA activity may have downregulated the *ste11* ^*+*^ expression and, in turn, *mat1-Mc* expression. This downregulation may cause a decrease in Tf2 antisense transcript production and this may lead to a marked SPAPB15E9.03c upregulation. This mechanism, in the reverse direction, can also explain the SPAC26A3.13c upregulation. On the other hand, the expression pattern of these retrotransposable elements, which are positively correlated with glucose availability, could suggest an answer about the relationship between retrotransposon expression and aging. It is well documented that there is a strong correlation between retrotransposition activity, genome instability, and aging and that the expression of many types of retrotransposons increases with age in a wide variety of organisms including yeast, *Drosophila*, and mammals (De Cecco et al., 2013, Li et al., 2013, Hu et al., 2014). However, whether this increase in expression is a consequence of aging or is directly involved in the aging process is not clear (Maxwell, 2016). As seen in many other organisms, calorie restriction extends *S. pombe* chronological lifespan when, for example, glucose levels were decreased 3 or 40 fold (Chen and Runge, 2009). In our study, RNAs were isolated from young mid-log phase cells. Thus, in concordance with the glucose availability, the elevated retrotransposon expression in young cells may directly contribute and determine the aging process under the condition of high glucose before these cells enter the stationary phase.

Glutathione S-transferases are a group of enzymes that catalyze the conjugation of glutathione with many exogenous and endogenous substances in the binding and detoxification of toxic compounds. In *S. pombe*, three different glutathione S-transferase coding genes, *gst1* ^*+*^ , *gst2* ^*+,*^ and *gst3* ^*+*^ , were identified (Veal et al., 2002). According to Kim *et al*., among them, only the expression of *gst3*+ was regulated by glucose or sucrose availability and its mRNA level was higher when a low concentration of glucose was present (Kim *et al*., 2004). They inferred that, as seen in *S. cerevisiae*, glucose depletion causes an increase in reactive oxygen species and this triggers *gst3* ^*+*^ expression. We observed that *gst2* ^*+*^ expression induced when glucose concentration was increased. Thus *gst2* ^*+*^ expression may also be regulated by glucose availability and induced when glucose concentration is increased.

hsp16+ is a small heat shock protein whose mRNA level is known to be induced by heat stress and its expression is activated through the transcription factor *atf1* ^*+*^ (Taricani et al., 2001). It was suggested that *hsp16* ^*+*^ expression may be controlled by nutritional conditions (Danjoh and Fujiyama, 1999). Indeed, the expression of HSP26 (*Saccharomyces cerevisiae* ortholog of *hsp16* ^*+*^ ), increased when glucose levels were low (Amoros and Estruch, 2001). In our study, the expression of *hsp16* ^*+*^ was downregulated consistently when the glucose concentration increased from both 3% and 5% to 8%. It was shown that the *hsp16* ^*+*^ expression repressed by *S. pombe* HIRA proteins which are histone chaperones that mediate nucleosome assembly and the activation of these proteins are also regulated by *atf1* ^*+*^ (Greenall et al., 2006). It is well known that glucose starvation is one of the environmental stresses and it seems that, under glucose-rich condition, the *hsp16* ^*+*^ expression is not necessary and suppressed by a mechanism which possibly involves *atf1* ^*+*^ . In our study, like that of *hsp16* ^*+*^ , the expression of *ssa1* ^*+*^ (or *hsp70* ^*+*^ ) which encodes another predicted heat shock protein was downregulated consistently when the glucose concentration increased. Similarly, it was reported that hsp70 mRNA levels in rat hepatocytes were significantly higher in caloric-restricted conditions than those observed when there was no such restriction (Heydari et al., 1993). On the other hand, it has long been known that Hsp70 counteracts protein aggregation along with Hsp104 and the association of Hsp104/Hsp70 is essential in longevity (Glover and Lindquist, 1998). In our study, the expression of hsp104 was downregulated when glucose level higher. Taken together, consistent downregulation of these heat shock proteins may cause a loss of cellular proteostasis which is considered as a hallmark of the aging process. Thus, excess sugar may deteriorate the cellular protective mechanism and make them less active just as seen during aging. Indeed, overexpression of these molecular chaperones ameliorates the age-associated decline in proteostasis and extends lifespan in *Caenorhabditis* and *Drosophila* (Walker and Lithgow, 2003, Morrow et al., 2004). 

*mam3*^*+*^ encodes an M-type-specific cell surface adhesion protein for conjugation between sexually flocculating cells (Mata and Bahler, 2006). Although the transcription of *mam3* ^*+*^ is induced under nitrogen starvation (Seike et al., 2013) and it is activated by mat1-Mc recruited Ste11, its expression seemed independent from mat1-Mc in our study. We observed that *mam3* ^*+*^ expression increased in both T2 and T3 conditions when compared to T1 condition while mat1-Mc expression downregulated in the T1 condition. This result raises the possibility of another function for *mam3* ^*+*^ other than adhesion. On the other hand, according to Liu et. al. (2015) flocculation activity in wild type *S. pombe* decreased with the lowering of glucose concentration. This result may support our finding that *mam3* ^*+*^ expression upregulated in response to elevated glucose concentrations but further studies are needed to clarify this situation.

When comparing the gene expression profile in T3 condition to those that T1, we saw that the expression of a group of genes related especially to kinetochore assembly, chromosome segregation, and mitotic progression markedly increased. These genes were *nbl1* ^*+*^ , *apc13* ^*+*^ , *spc19* ^*+*^ , *tea2* ^*+*^ , *mis6* ^*+*^ , *mis18* ^*+*^ , *mis15* ^*+*^ . Mis proteins function as a structural core and regulator of the kinetochore (Muller and Almouzni, 2017). Together with some other subunits, Mis18 complex is required for the centromeric localization of the Mis6-Mis15 complex and Cnp1 during the cell cycle (Takahashi et al., 2005). Nbl1 is a borealin homolog gene and a subunit of a chromosome passenger complex (CPC) which is required to destabilize and repair inaccurate kinetochore attachments. Binding of borealin to nucleosome is essential for this complex to associate with the chromosome and for the error-free chromosome segregation (Abad et al., 2019). Apc13 is a member of the Anaphase Promoting Complex (APC) which maintains genomic stability and initiates the transition from metaphase to anaphase and coordinate chromosome segregation in mitosis by routing specific proteins for degradation (Zhang et al., 2016). Spc19 is a component of the DASH complex which forms a ring around a microtubule and responsible for the correct chromosome segregation and bipolar attachment of sister chromatids on the mitotic spindle (Sanchez-Perez et al., 2005). Tea2 encodes a kinesin that regulates microtubule growth by transporting regulatory factors to the growing microtubule tips (Busch et al., 2004). Taken together, the increase in the expression of these genes may indicate a cellular activity that provides error-free chromosome segregation. Glucose detection results in the activation of the cAMP-dependent protein kinase A (PKA) in *S. pombe* and PKA coordinates cell proliferation depending on glucose status (Hoffman, 2005). Although many studies suggest that PKA is an inhibitor of the APC, the interaction between the cAMP-PKA pathway and the kinetochore is thought to be more complex (Ma et al., 2012). On the other hand, it is thought that the APC may be involved in enhancing genomic stability through repairing DNA damage that occurs during chromosome segregation but this process has not been fully elucidated (Harkness, 2018). Recent works revealed a connection between glucose availability and kinetochore activity and bipolar spindle formation during mitosis (Shah et al., 2019, Tanabe et al., 2020). Accordingly, higher glucose levels destabilize kinetochore attachment and weaken kinetochore-microtubule binding through phosphorylation of Dam1 which is *S. cerevisiae* ortholog of Spc19. Growth defects and monopolar spindle formation in a kinesin-5 mutant was partially rescued by *pka1* ^*+*^ deletion or glucose limitation in *S. pombe*. Under glucose-rich condition, cells may tolerate higher rates of mitotic infidelity to reproduce faster. On the other hand, the expression of these mitotic regulators may have increased to cope with the genome instability resulting, for example, from increased retrotransposon activity due to the presence of excess glucose. The relationship between nutrient availability and mitotic regulators has only recently been studied. Thus, further investigations are needed to clarify this link.

**Figure 6 -:**
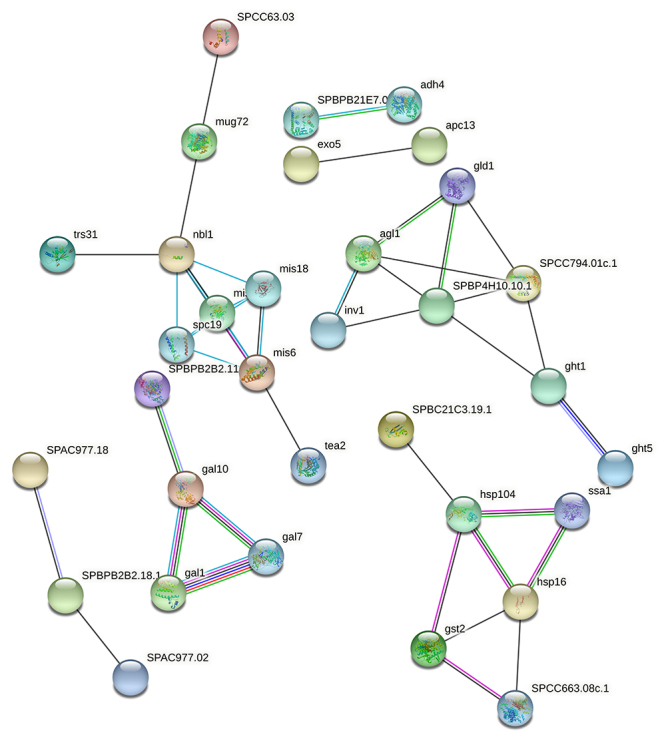
The putative interaction map of proteins that encoded by all genes whose expression were significantly changed acording to glucose availability. Network was created through STRING website (ver. 11.0). Interaction sources included experiments, databases, co-expression, neighborhood, gene fusion and co-ocurrence. Minimum required interaction score was set as 0.400. Detailed explanation is given in the text.

The other group whose expression in T3 condition at least two-fold increased when compared to T1 includes three transcription regulators or coactivators. One of them, *spt3* ^*+*^ is a subunit of the SAGA (Spt-Ada-Gcn5 acetyltransferase) complex which acts as a general cofactor, chromatin remodeler, and sets the accessibility for DNA transcription (Helmlinger et al., 2008). *taf3* ^*+*^ is predicted to be a subunit of TFIID, a complex composed of TATA-binding protein (TBP) and TBP associated factors (TAFs) (Matangkasombut et al., 2004) The third one, *med13* ^*+*^ is the component of Cdk8 module of the multiprotein Mediator complex which is a central integrator of transcription. In addition to the targeting of CDK8 by several different intracellular signaling pathways, the loss of Med13 may lead to the early entry of the cell into mitosis, suggesting that this protein also regulates the mitotic progression (Banyai et al., 2017). On the other hand, the components of TFIID, SAGA complex, and Mediator are known to interact with each other in certain cellular processes, but there is much unknown about this relationship (Donczew et al., 2020, Papai et al., 2020). It is reported that the SAGA complex is a direct target of nutrient‐sensing pathways and regulates, for example, switching from proliferation to sexual differentiation when the glucose level is low (Helmlinger *et al*., 2008, Laboucarie et al., 2017). On the other hand, the deletion of the SPT3 gene was shown to lead to a severe decrease in Ty1 retrotransposon transcription in *S. cerevisiae* (Winston et al., 1984, Curcio et al., 2015). Similarly, the marked increase in the retrotransposable element expression depending on elevated glucose levels that we observed in this study may be explained partly by the increase in *spt3* ^*+*^ expression. Interestingly, overexpression of MED13 improved glucose tolerance and insulin sensitivity in mice cardiomyocytes (Dannappel et al., 2018). This effect of MED13 results from increased energy expenditure and regulation of numerous genes involved in energy balance (Grueter et al., 2012). Besides, the knockdown of MED13 increases susceptibility to obesity by regulating Wingless in Drosophila (Lee et al., 2014). Thus, the induced expression of med13 in this study may contribute to regulating energy balance in the presence of excess glucose. Taken together, our findings are consistent with these results and may help to clarify the link between glucose availability and the coactivators. The putative interaction map of proteins that encoded by all genes whose expression were significantly changed acording to glucose availability was shown in Figure 6.

Finally, the expression of a group of genes that can be classified unassigned, sequence orphan, dubious, or conserved fungal and whose functions are unknown were also downregulated or upregulated in response to elevated glucose levels. Importantly, while the expression of SPBC660.05 was significantly reduced, SPAC959.06c was upregulated in response to increasing glucose concentration. These genes are predicted to be conserved fungal proteins and because the consistent change in their expression suggests a role in glucose metabolism they can be the candidates for further studies. 

## Conclusions

Besides being a universal energy source, glucose also functions as a signal molecule in cells. Therefore, it affects many cellular processes such as division, proliferation, and aging. From this point of view, we conducted a genome-wide analysis to clarify how glucose presence affects genome-wide gene regulation. Our results indicated that excess glucose, as expected, downregulated many genes that function in carbohydrate metabolism. On the other hand, we found that there were significant and consistent changes in the expression of genes encoding retrotransposons, heat shock proteins, transcription regulators, and proteins that provide genomic stability. We suggest some complementary roles and possible interactions of these gene expression changes that can explain the possible pro-aging effect of excess glucose. These suggestions may contribute to the findings obtained in recent years regarding the relationship between glucose availability and diverse cellular mechanisms. We also observed significant changes in the expression of some genes that have not yet been well characterized in *S. pombe*. Thus, their roles in glucose metabolism and glucose-dependent cellular response can be the subject of future studies. We hope that the large scale analysis we have performed will serve as the basis for more detailed studies on the relationship between glucose availability and aging. 

## Supplementary material

The following online material is available for this article:

Table S1 - The quantitative change in the expression of individual genes depending on the glucose concentration.
