# Supplementary material for: Transcriptome sequencing and screening of genes related to glucose availability in Schizosaccharomyces pombe by RNA-seq analysis
Source: Genet Mol Biol. 2021 Aug 27;44(3):e20200245. doi: 10.1590/1678-4685-GMB-2020-0245 (PMC8404550; doi:10.1590/1678-4685-GMB-2020-0245)
Supplement: Table S1 - [file 1415-4757-GMB-44-3-e20200245-s1.pdf]

Genetics and Molecular Biology

https://doi.org/10.1590/1678-4685-GMB-2020-0245

Supplementary Material to "Transcriptome sequencing and screening of genes related to glucose availability in *Schizosaccharomyces pombe* by RNA-seq analysis"

Table S1. The quantitative change in the expression of individual genes depending on the glucose concentration.

**Systematic ID / Gene name**	**Fold change in gene expression according to the glucose concentration**		
	Expression in 3% compare to %5	Expression in 8% compare to 3%	Expression in %8 compare to 5%
SPAPB24D3.10c (maltose alpha-glucosidase Agl1)	-2,15	-2,09	-4,24
SPCC548.07c (hexose ransmembrane transporter Ght1)		-3,00	-4,07
SPCC1235.14 (hexose transmembrane transporter Ght5)			-2,26
SPBPB2B2.13 (galactokinase Gal1)		-2,37	-2,65
SPBPB2B2.10c (galactose-1-phosphate uridylyltransferase Gal7)		- 2,21	-2,62
SPBPB2B2.12c (UDP-glucose 4-epimerase/aldose 1-epimerase Gal10)			-2,71
SPCC1223.03c (glycerol-3-phosphate dehydrogenase Gut2 (predicted))		-3,07	-3,43
SPAC13F5.03c (mitochondrial glycerol dehydrogenase Gld1)		-3,78	- 4,66
SPCC794.01c (glucose-6-phosphate 1-dehydrogenase Zwf2 (predicted)			-2,61
SPBPB2B2.11			-2,61
SPCC191.11 (external invertase, beta-fructofuranosidase)			-2,88
**Systematic ID / Gene name**	**Fold change in gene expression according to the glucose concentration in SD medium**		
	Expression in 3% compare to %5	Expression in 8% compare to 3%	Expression in %8 compare to 5%
SPAPB15E9.03c (retrotransposable element/transposon Tf2-type)	-9,71		
SPAC26A3.13c (retrotransposable element/transposon Tf2-type)			+2,24
SPBC3E7.02c (heat shock protein Hsp16)		-3,17	-2,06
SPBC16D10.08c		- 2,13	

(heat shock protein Hsp104)			
SPAC13G7.02c<br>(heat shock protein Ssa1 (predicted))		-2,75	-2,29
SPBC23G7.09, mat1-Mc<br>(mating type m specific polypeptide)	+8,64		
SPCC965.07c<br>(glutathione S-transferase Gst2)		+2,68	+ 2,03
SPAP11E10.02c<br>(cell agglutination protein Mam3)		+2,33	+1,52
SPBC685.02<br>(mitochondrial single stranded DNA specific 5'-3' exodeoxyribonuclease Exo5 (predicted))		+2.51	
SPCC61.02<br>(SAGA complex subunit Spt3)		+2,45	
aes1<br>enhancer of RNA-mediated gene silencing)			-2,29
SPAC823.06<br>(transcription factor TFIID complex subunit Taf3 (predicted))		+ 2,43	
SPBC1718.05<br>(TRAPP complex subunit Trs31 (predicted))		+2,41	
SPBP4H10.09<br>(transcription factor Rsv1)			-2,82
SPAC1071.04c<br>(signal peptidase subunit Spc2 (predicted))		+2,40	
SPBP4H10.10<br>(rhomboid family protease)			-2,40
**Systematic ID / Gene name**	**Fold change in gene expression according to the glucose concentration in SD medium**		
	Expression in 3% compare to %5	Expression in 8% compare to 3%	Expression in %8 compare to 5%
SPCC63.03<br>(DNAJ domain protein, DNAJC11 family)		+2,40	
SPAC5H10.06c<br>(aldo/keto reductase, predicted calcium channel regülatör)		+2,34	
SPBC29A3.03c<br>(GID complex ubiquitin-protein ligase E3 subunit Gid2/Rmd5 (predicted))		+2,27	
SPCC663.08c<br>(short chain dehydrogenase)		+2,17	
SPAC869.02c<br>(nitric oxide dioxygenase Yhb1)		+2,15	
SPBC887.06c<br>(sorting nexin Snx3 (predicted))		+2,14	
SPCC1020.01c<br>(P-type proton ATPase, P3-type Pma)		+2,08	
SPAC12G12.09<br>(conserved fungal protein associated with stress granüle)		+2,01	
SPBC359.04c		-3,45	

(cell surface glycoprotein (predicted), DIPSY family)			
SPBPB2B2.05<br>(peptidase family C26 protein)		-2,32	
SPBC21C3.19<br>(SBDS family protein Rtc3 (predicted))		-2,18	
SPBC725.12<br>(Borealin homolog Nbl1)		+4,32	
SPBC28E12.01c<br>(anaphase-promoting complex TPR lobe accessory factor Apc13)		+3,13	
SPCC1902.02<br>(Meiotically Upregulated Gene, mug72)		+2,5	
SPCC1223.15c<br>(DASH complex subunit Spc19)		+2,34	
**Systematic ID / Gene name**	**Fold change in gene expression according to the glucose concentration in SD medium**		
	Expression in 3% compare to %5	Expression in 8% compare to 3%	Expression in %8 compare to 5%
SPBC1604.20c<br>(kinesin family plus-end microtubule motor Tea2)		+2,27	
SPAC1687.20c<br>(kinetochore protein, CENP-I ortholog Mis6)		+2,05	
SPCC970.12<br>(kinetochore protein Mis18)		+2,02	
SPAC589.02c<br>(mediator complex subunit Med13)		+2,00	
SPBP22H7.09c<br>(kinetochore protein, CENP-N ortholog Mis15)		-2,64	
SPAC750.04c<br>(S. pombe specific 5Tm protein family)	-2,54		
SPAC959.06c<br>(conserved fungal protein)		+2,95	+2,02
SPBC660.05<br>(WW domain containing conserved fungal protein)		-4,44	-4,40
SPAC977.18<br>(conserved fungal protein)	-2,41		-2,23
SPCC576.19c<br>(dubious)			+2,29
SPBPB2B2.18<br>(*Schizosaccharomyces* specific protein)	-2,75		
SPBPB21E7.04c<br>(human COMT ortholog 2)	-2,10		
SPCC1235.18<br>(dubious)		-2,32	
SPCP20C8.02c<br>(*S. pombe* specific UPF0321 family protein 1)			+2,01
SPAC959.11<br>(*Schizosaccharomyces* specific protein)			-12,44

SPAC2F3.07c<br>(*Schizosaccharomyces pombe* specific protein)	-2,05		
SPAC4F8.08<br>(*Schizosaccharomyces pombe* specific protein Mug114)	-2,69		
